# Evaluation of Liposomal and Conventional Amphotericin B in Experimental Fungal Keratitis Rabbit Model

**DOI:** 10.1167/tvst.8.3.35

**Published:** 2019-06-06

**Authors:** Anup Kumar Ghosh, Shivaprakash Mandya Rudramurthy, Amit Gupta, Hansraj Choudhary, Shreya Singh, Anchal Thakur, Manu Jatana

**Affiliations:** 1 Department of Medical Microbiology, PGIMER, Chandigarh, India; 2 Department of Ophthalmology, PGIMER, Chandigarh, India

**Keywords:** amphotericin b, liposomal amphotericin b, *Aspergillus flavus*, *Candida albicans*, keratitis, rabbit model

## Abstract

**Purpose:**

We evaluate the efficacy of liposomal amphotericin (Fungisome) compared to conventional amphotericin (AMB) for the treatment of fungal keratitis (FK) in an experimental rabbit model.

**Methods:**

FK was induced in 48 New Zealand White rabbits using *Aspergillus flavus* and *Candida albicans* (24 rabbits each). Rabbits were divided into four groups: 0.1% and 0.05% Fungisome-, and 0.1% AMB-treated groups, and one untreated control group. Clinical scores were recorded throughout the study while fungal burden was estimated by corneal button culture on day 19 (study endpoint).

**Results:**

A statistically significant improvement in clinical score was seen on day 11 in the 0.1% and 0.05% Fungisome versus untreated groups (13.91 and 14.4 vs. 19.3; *P* < 0.001) in the *A. flavus* model, and on day 9 in the 0.1% Fungisome-treated versus untreated groups (12.96 vs. 14.2; *P* = 0.006) in the *C. albicans* model. At endpoint, the mean clinical scores of the untreated controls, and the 0.1% and 0.05% Fungisome-, and 0.1% AMB-treated groups were 20 ± 1.4, 5.33 ± 1.85, 9.66 ± 2.41, and 8.16 ± 1.95, respectively, in the *A. flavus* model and 15.85 ± 1.87, 3.08 ± 1.31, 4.21 ± 1.370, and 4.13 ± 1.38, respectively, in the *C. albicans* model. Conjunctival hyperemia score was higher in the 0.1% AMB- versus 0.1% Fungisome-treated groups (1.33 vs. 0.5, *P* = 0.452). Lowest fungal burden in both models was seen in the 0.1% Fungisome-treated groups.

**Conclusions:**

Clinical improvement was observed with Fungisome relative to untreated controls. However, no statistically significant differences in outcomes were observed between animals treated with Fungisome and AMB. Although the results are encouraging, future studies in humans are warranted.

**Translational Relevance:**

FK is a leading cause of corneal blindness and is on the rise especially in developing countries. Despite the availability of various antifungal agents, heterogeneous treatment outcomes are seen due to lack of a standardized treatment regimen for FK. Although the use of liposomal AMB has been substantiated by clinical evidence in systemic infections, to our knowledge there are no in vivo studies evaluating the role of topical liposomal versus conventional formulation in FK. Our study investigated the efficacy and toxicity profile of liposomal versus conventional formulation of AMB in an experimental rabbit FK model.

## Introduction

Fungal keratitis (FK) is a potentially serious cause of visual impairment worldwide accounting for 50% to 60% of microbial keratitis in developing countries.^1^,^2^ More than 105 species of fungi have been reported to cause keratitis, with *Aspergillus, Fusarium*, and *Candida* being the most frequent and together responsible for 44% of all corneal ulcer cases.^3^ Prompt diagnosis and treatment with appropriate antifungal agents is crucial for the management of FK. Several topical antifungal agents are effective, including natamycin azoles derivatives, such as fluconazole, voriconazole, itraconazole, and amphotericin B (AMB).^4^–^6^

AMB is a broad-spectrum, polyene antifungal active against genus *Candida, Aspergillus, Penicillium, Cryptococcus*, and *Mucor*. It acts by binding to the sterols in the fungal cell wall to form pores, which alter the cell permeability and osmotic gradient resulting in eventual cell death. In addition, AMB has been shown to increase the formation of intracellular reactive oxygen species causing oxidative stress, which promotes its fungicidal action.^7^ The unique anatomic and physiologic barriers at the ocular surface, such as the tear barrier and corneal tight junctions, hamper drug penetration and achievement of adequate bioavailability of this topically applied drug. In addition, conventional AMB has side effects of ocular irritation, superficial corneal erosions, and green discolouration.^8^

To overcome these limitations, liposomal amphotericin (L-AMB), a lipid-associated formulation, is used to treat FK. Since liposomal formulation slows down the ocular drug clearance and acts as a drug reservoir, AMB concentrations within the eye are constant with L-AMB. One study reported the ocular bioavailability of liposomal antifungals to be equal to or better than that of nonliposomal ones.^9^ This was supported by Pleyer et al.^10^ in an animal study suggesting that topically delivered L-AMB provides stable corneal drug levels and lower ocular toxicity.

In India, L-AMB has been developed indigenously and is being marketed as Fungisome (Lifecare Innovations Pvt. Ltd., Gurgaon, India). Fungisome contains AMB in small unilamellar phosphatidylcholine and cholesterol (ratio 7:3) liposomes.^11^ Although, clinical trials reveal more than 90% efficacy and negligible toxicity in systemic fungal infections treated with Fungisome, its efficacy in FK remains to be evaluated.^12^ Thus, we planned this study to elucidate the efficacy of Fungisome compared to conventional AMB for the treatment of *A. flavus-* and *C. albicans*-induced FK in experimental rabbit model.

## Materials and Methods

A total of 48 New Zealand white rabbits (age, 4-6 weeks; weight, 2.5–3.5 kg) were used for the study. The study protocol was approved by the institutional ethical committee, and was conducted in accordance with the Association for Research in Vision and Ophthalmology (ARVO) Statement for Use of Animals in Ophthalmic and Visual Research. Rabbits were anesthetized with intramuscular ketamine (35 mg/kg) and xylazine (5 mg/kg). Corneal anesthesia was established using topical 0.5% proparacaine eye drops. FK was induced only in the right eye of every rabbit in this study. All rabbits were assigned numbers 1 to 48 in a random order. A starting point was randomly selected and numbers were grouped by picking every other number into two groups (*A. flavus* and *C. albicans* keratitis model groups with 24 rabbits each). In each group, infected rabbits were similarly divided into four groups with six rabbits each using a table of random numbers.

### Inoculum Preparation

Isolates of *A. flavus* (ATCC 204304) and *C. albicans* (ATCC 90028) were used in the study. *A. flavus* was grown on potato dextrose agar (PDA) slant at 30°C for 3 to 10 days. A stock inoculum suspension was prepared in 3 to 4 mL sterile saline with 0.1% Tween 80. It then was vortexed and filtered through two layers of sterile gauze to remove hyphal fragments. The suspension then was centrifuged at 10,000*g* for 5 minutes and the pellet was rediluted in sterile normal saline to get the concentration of 10^6^ conidia/mL.

Similarly, *C. albicans* was grown on Sabouraud's dextrose agar (SDA) plate at 35°C for 24 hours. Five colonies were suspended in 5 mL 0.85% sterile saline to obtain a final concentration of 10^6^ cells/ mL.

### Antifungals

Fungisome and conventional AMB (Lifecare Innovations Pvt. Ltd) were used in this study. Fungisome was sonicated in a water bath at 25°C for 45 minutes before administration and both antifungals were stored in dark bottles and at 2° to 8°C.

### Induction of FK

#### *A. flavus*-Induced Model

To develop the *A. flavus*-induced keratitis model in rabbits, contact lens and intrastromal mode of infection were evaluated and the intrastromal injection method was chosen, as it was less cumbersome, more cost-effective and equally effective as the contact lens model (data not shown). A total of 24 rabbits were anesthetized and 20 μL inoculum was injected intrastromally using a bent 30-gauge insulin needle under slit-lamp guidance. Development of FK was observed on day 5 after inoculation following which treatment was initiated. For antifungal treatment, the rabbits were divided into four groups of six infected rabbits each and treated with 0.1% and 0.05% Fungisome, 0.1% conventional AMB, and normal saline (untreated controls). Sequentially numbered, opaque drug containers with identical appearance were used for drug administration to reduce performance bias. Drug was instilled as eye drops (1 drop of 20 μL) into the infected eye. Topical application was repeated every 1 hour for 9 hours for 19 consecutive days. The infected rabbit eyes were evaluated and graded clinically every 48 hours until day 19 with the help of a slit-lamp examination by an ophthalmologist. Clinical scores were tabulated for each group and the mean was calculated.

#### *C. albicans*-Induced Model

Since the suspension of *C. albicans* cells was very viscid, the intrastromal route was not feasible and the contact lenses model was chosen. All 24 rabbits were anesthetized and the nictitating membrane of the right eye was removed by sharp dissection. A 7-mm filter paper disk moistened with 99% isopropyl alcohol (Merck, Kenilworth, NJ) was placed on the center of the cornea for 30 seconds and the corneal epithelium was removed. The eye was rinsed with sodium lactate solution to remove any remaining traces of isopropyl alcohol. A grid pattern of abrasions was made on the central cornea. The fungal inoculum was transferred to the denuded cornea using a large-bore pipette tip and the inoculum was retained in the cornea by placing the sterile contact lens (diameter 14.0 mm; PureVision; Bausch and Lomb, Waterford, Ireland). To prevent contact lens extrusion, tarsorrhaphy was performed using 5-0 Mersilk suture to close the lids. All procedures were performed aseptically. The eyes were examined after 48 hours by removing contact lens and reexamined subsequently every 48 hours. Infected rabbits were divided into four groups, treated and followed as described previously in the *A. flavus* keratitis model.

### Clinical Evaluation and Scoring

Clinical examination and scoring was done on days 1, 3, and 5 after inoculation to determine the development of FK. FK was observed in all rabbits after 5 days. Treatment was started on day 5 and rabbits were examined on days 7, 9, 11, 13, 15, and 19 after inoculation. Parameters, such as conjunctival hyperemia, corneal clouding, corneal infiltration (measured as size of epithelial defect in mm), corneal neovascularization and hypopyon level, in each rabbit were evaluated and noted. Clinical score was determined with a brief modification by following the Schreiber scoring system.^13^ In brief, circumcorneal congestion was graded as follows: 0, no; 1, mild; 2, medium; and 3, high grade congestion. Corneal clouding was graded as follows: 0, no clouding or clear cornea; 1, mild clouding; 2, corneal clouding in two quadrants of the cornea; 3, total corneal clouding. The extent of corneal neovascularization was graded as 0, no; 1, mild (up to 2 mm from the limbus); 2, medium (>2 mm from the limbus); and 3, high (until the center of the cornea) neovascularization. The corneal infiltrate and hypopyon diameter were measured in millimeters using a portable slit-lamp biomicroscope. The rabbits cornea was examined until day 19 after inoculation since corneal perforation was observed in the untreated control group. Drug toxicity in all treated groups was assessed by the presence of hyperemia in the lower forniceal conjunctiva. The list of treatment assignment was made by a statistician independently. Sequentially numbered, opaque drug containers with identical appearance were used for treatment. The drugs were administered by an ophthalmologist and clinical evaluation was performed by two different masked ophthalmologists.

### Determination of Fungal Burden

On day 19, the corneal button was aseptically removed (treated and untreated), transferred to 1 mL sterile phosphate buffered saline (PBS), and homogenized. Serial dilutions were plated on SDA to determine the fungal load. Plates were incubated for 2 days at 37°C for *C. albicans* culture and 5 days at 28°C for *A. flavus* culture.

### Statistics

Non-parametric repeated measures tests (Friedman test) in SPSS software version19 and Graph Pad in Stat 4 software were used to analyze the changes in clinical parameters over time. The *P* value was calculated by using 1-way analysis of variance followed by Bonferroni to compare all pairs of columns and a value of >0.5 was considered significant.

## Results

### Results of Clinical Evaluation and Scoring

In the *A. flavus*-induced FK model, the baseline mean clinical score in the four groups on day 5 after inoculation was 13.2, 15.16, 14.7, and 14.34 in the 0.1% and 0.05% Fungisome, conventional AMB, and untreated control groups, respectively (*P* = 0.364).

**Figure 1:**
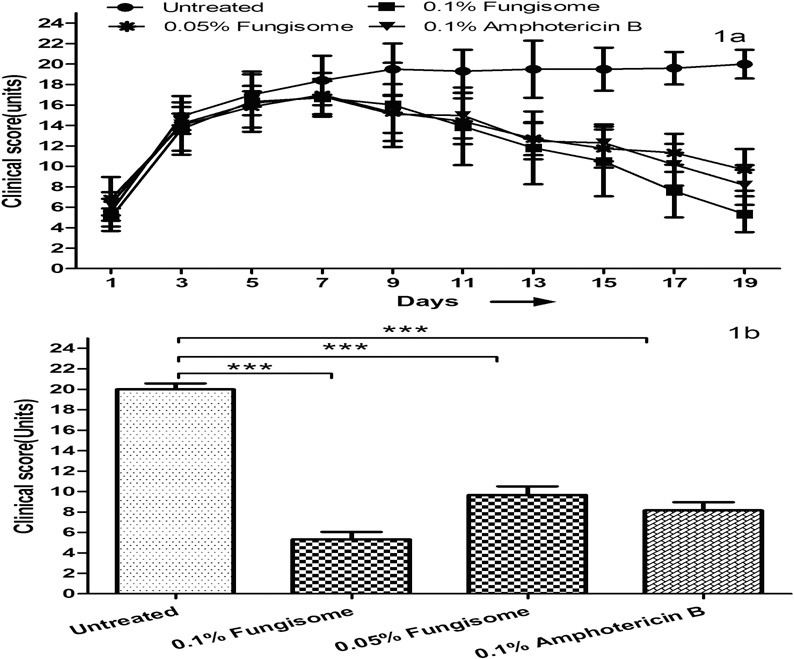
(a) Improvement in clinical scores in A. flavus-induced FK in rabbits treated with 0.1% and 0.5% Fungisome, and 0.1% Amphotericin B compared to untreated controls. (b) Clinical scores in A. flavus-induced keratitis in rabbits treated with different drugs in compared to untreated controls on day 19. All three treated groups are significantly different (***P < 0.001) compared to the untreated group.

An increase in the mean clinical score was observed on day 7 after inoculation (i.e., 2 days after initiation of treatment; Fig. 1a). Although clinical improvement began on the day 9 after inoculation (i.e., 4 days after initiation of treatment) in all treated groups, the difference was not significant compared to the untreated controls. We observed a statistically significant improvement in mean clinical score of groups treated with 0.1% (mean ± standard deviation [SD], 13.91 ± 3.8) and 0.05% (mean 14.4 ± 2.41) Fungisome compared to untreated controls (mean 19.3 ± 3.1) from day 11 onwards after inoculation (*P* < 0.001; Fig. 1a). Although a reduction in mean clinical score was noted on day 11 in the 0.1% conventional AMB-treated group, it was not statistically significant (*P* = 0.39). At the study endpoint (day 19), the mean overall clinical score was 20 ± 1.4, 5.33 ± 1.85, 9.66 ± 2.41, and 8.16 ± 1.95 in the untreated controls, 0.1% and 0.05% Fungisome-treated groups, and 0.1% conventional AMB-treated group, respectively (Fig. 1b). All three treated groups were significantly different (*P* < 0.001) compared to the untreated group; however, there was no significant difference among the three treated groups on day 19.

**Figure 2:**
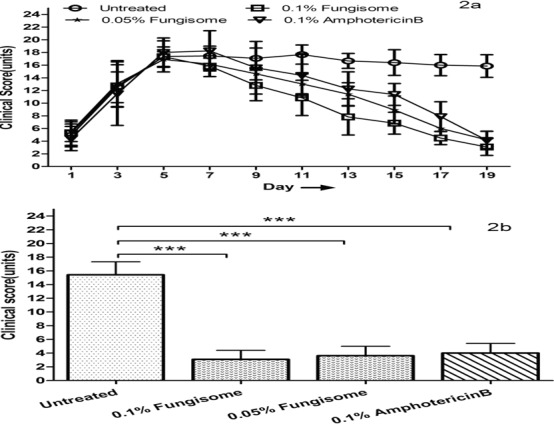
(a) Improvement in clinical scores in C. albicans-induced keratitis in rabbits treated with 0.1% and 0.5% of Fungisome, and 0.1% Amphotericin B compared to untreated controls. (b) Clinical scores in C. albicans-induced keratitis in rabbits treated with different drugs compared to untreated controls on day 19. All three treated groups are significantly different (***P < 0.001) compared to the untreated group.

In the *C. albicans*-induced keratitis model, the mean clinical scores at baseline were 17.37, 17.33, 16.88, and 18 in the untreated, 0.1% and 0.05% Fungisome, and 0.1% AMB-treated group, respectively (*P* = 0.243; Fig. 2a). Statistically significant clinical improvement was seen only in the 0.1% Fungisome-treated compared to the untreated groups (12.96 vs. 14.2; *P* = 0.006 ) on day 9 after inoculation. Subsequently, a statistically significant improvement in mean clinical score (*P* < 0.01) was noted in all treated groups from day 11 after inoculation until day 19 (study endpoint). At the study end (day 19) the clinical score of the 0.1% (mean 3.08 ± 1.31) and 0.05% (mean 4.21 ± 1.37) Fungisome-treated group, and the 0.1% AMB-treated group (mean 4.13 ± 1.38) was lower than that in the untreated group (mean 15.85 ± 1.87; *P* < 0.0001; Fig. 2b). Although the 0.1% Fungisome-treated group had the least clinical scores at study end, there was no significant difference among the three treated groups.

**Figure 3:**
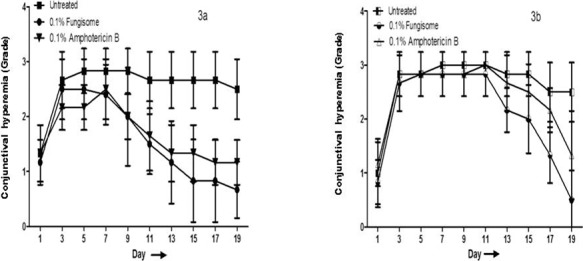
Treatment effect and comparison of conjunctival hyperemia between 0.1% Fungisome- and 0.1% Amphotericin B-treated group of A. flavus- (a) and C. albicans- (b) induced models.

Since the results of clinical scoring were better with 0.1% compared to 0.05% Fungisome, toxicity assessment was performed only for 0.1% Fungisome. In the *A. flavus* model, the trends of conjunctiva hyperemia showed a reduction in both treated groups versus the untreated controls (Fig. 3a). Hyperemia in the 0.1% Fungisome-treated and 0.1% AMB-treated groups declined (compared to the untreated group) until day 13, with a mean hyperemia score of 1.16 ± 0.75 in the 0.1% Fungisome- and 1.33 ± 0.51 in the 0.1% AMB-treated groups. On day 19, the conjunctival hyperemia in the lower fornix was higher in the AMB-treated compared to the 0.1% Fungisome-treated groups (1.0 vs. 0.6 ± 0.5; *P* < 0.001).

Similarly, in the *C. albicans*-induced model, the mean score for forniceal conjunctival hyperemia was similar among all groups until day 11. On day 13, a decrease in hyperemia was noted in both treated groups, which was seen until day 19 (Fig. 3b). At study end, the hyperemia score was higher in the 0.1% AMB- (mean score, 1.33 ± 0.81) compared to the 0.1% Fungisome- (mean score = 0.5 ± 0.33) treated groups. However, this difference was not statistically significant (*P* = 0.452).

### Assessment of Corneal Infiltration

**Figure 4:**
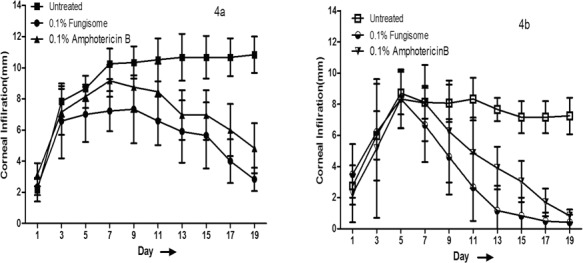
Treatment effect and comparison of corneal infiltrations between 0.1% Fungisome and 0.1% Amphotericin B-treated groups of A. flavus- (a) and C. albicans- (b) induced models.

Corneal infiltration was reduced by day 19 of treatment compared to the untreated group in both keratitis models. The size of corneal infiltration over time in the *A. flavus* keratitis model is depicted in Figure 4a. A decline in infiltration size was observed on day 7 in the 0.1% amphotericin-treated group and on day 9 in the 0.1% Fungisome-treated group, while no decline was seen in the untreated controls. At the study endpoint, corneal infiltration size in the 0.1% AMB-treated group was higher than in the 0.1% Fungisome-treated group (3.83 vs. 2.5 mm; *P* = 0.213).

In the *C. albicans*-induced keratitis model, a reduction in corneal infiltrates was evident immediately after initiation of treatment (i.e., day 5) with a sustained decline in the treated groups compared to the untreated controls (Fig. 4b). Infiltration size in the 0.1% AMB-treated group was higher than in the 0.1% Fungisome-treated group on day 19 (mean infiltration size, 0.83 vs. 0.41; *P* = 0.178).

### Result of Fungal Burden Evaluation

The results are shown in the Table. The total number of colonies recovered from the *A. flavus*-infected cornea was highest in untreated controls (376.6 ± 68.08) followed by the 0.05% Fungisome- (129.83 ± 31.99) and 0.1% AMB- (68.33 ± 9.6) treated groups, and lowest in the 0.1% Fungisome-treated group (19.16 ± 11.03; *P* < 0.001).

**Table:** Total Number of Colonies Recovered From A. flavus- and C. albicans-Infected Corneas, Mean and SD of Each Group Calculated

	Untreated	0.1% Fungisome	0.05% Fungisome	0.1% AMB
*A. flavus* keratitis model				
Mean	376.66	39.16	129.83	68.33
SD ±	68.08	11.03	31.99	9.58
*C. albicans* keratitis model				
Mean	300.83	22.5	79.83	23.33
SD ±	67.17	7.42	25.36	8.59

In the *C. albicans*-infected cornea, the fungal burden was highest in untreated controls (300.83 ± 67.17) followed by the 0.05 Fungisome-treated group (79.8 ± 8.59), while the fungal burden in the 0.1% AMB and 0.1% Fungisome groups was the lowest at 23.3 ± 8.59 and 22.5 ± 7.42, respectively (*P* < 0.001).

## Discussion

FK is a major cause of corneal blindness, especially in developing countries, where over half of the cases of keratitis are reported to be of fungal etiology.^14^,^15^ Clinical outcome depends on prompt diagnosis and treatment with appropriate antifungal agents. Poor corneal penetration of antifungal agents, limited efficacy of currently available antifungals, drug resistance, and emerging fungal pathogens affect treatment outcome.^16^ Antifungal treatment duration especially in cases of FK caused by filamentous fungi is 8 to 12 weeks, often requiring longer treatment due to slow clinical improvememnt.^17^ Although the first line of treatment for FK is natamycin, recent reports of increased minimal inhibitory concentration of various fungal pathogens and poor coverage of against *Candida* species warrants use of alternative antifungal agents.

The antifungal agent, AMB, is active against a broad spectrum of fungi (filamentous and yeast forms) and has been used successfully to treat FK.^18^–^20^ It has been administered through various routes, including systemic, intravenous, topical, intracorneal, intracameral, and subconjunctival routes. Unlike systemic administration, topical application of AMB in the eye has better corneal penetration.^21^,^22^ Topical application of conventional AMB has lower absorption through mucosa. Additionally, since AMB has an affinity for fungal ergosterol, it also can bind to structurally similar cholesterol in human cells and damage them, resulting in side effects of hyperemia, itching, and dryness in mucosa and skin.^6^ To circumvent the limitation of conventional AMB preparations, several lipid-based AMB formulations have been developed. These include AMB lipid complex (ABLC), L-AMB, and AMB colloidal dispersion (ABCD), which differ considerably in their pharmacokinetic parameters. When given systemically, L-AMB is known to have better bioavailability with lower renal toxicity and infusion-related side effects than conventional AMB.^23^ Better tolerability also has been seen in topical formulation (primarily in skin infections) of L-AMB compared to AMB.^24^ Although AMB has been used topically to treat FK,^10^,^18^ to our knowledge no report has investigated the efficacy and toxicity profile of L-AMB over conventional AMB. We designed an experimental FK rabbit model to compare L-AMB (0.05% and 0.1% Fungisome) with conventional AMB (0.1% concentration) to treat *A. flavus-* and *C. albicans*-induced keratitis. We chose the two concentrations of Fungisome to check if a lesser dose was associated with similar clinical action and a lower toxicity profile. In addition, eye drops based on 0.1% L-AMB can be stored for only up to 1 week after reconstitutions, while a longer shelf life at ambient temperature has been seen with a 0.05% ophthalmic preparation.^25^ Thus, a similar clinical action, lower toxicity, and longer shelf life would make this preparation extremely convenient.

In our study, all rabbits (both models) had keratitis 5 days after inoculation and had similar clinical scores at baseline. In the *A. flavus* keratitis model, clinical improvement began at day 7 on treatment with AMB and Fungisome. However, improvement was statistically significant after 11 days both Fungisome groups (0.05% and 0.1%). In the *C. albicans*-induced keratitis model, clinical improvement appeared immediately after treatment in all groups. However, it was statistically significant earlier in the 0.1% Fungisome-treated group than in the other groups (days 9 vs. 11). Thus, overall an earlier response to treatment was seen in *C. albicans-* compared to *A. flavus*-induced FK. This could be due to better action of amphotericin, better penetration of drug, and lower degree of tissue damage in *C. albicans-* compared to *A. flavus*-induced keratitis. Endophthalmitis due to molds, such has *Aspergillus* species, has been more aggressive than that due to *Candida* species with faster progression and worse outcome.^26^ Our findings suggest a similar trend with FK. Interestingly, in a study in Danish patients, FK due to *Aspergillus* had higher incidences of complications, but better response with antifungal medications versus FK due to *Candida* species.^27^ However, in their study, although FK due to molds was treated predominantly with AMB (83% patients), it was used only in 8% of *Candida* FK cases. Future studies evaluating the clinical characteristics and variable outcomes of FK in mold versus yeast infections in humans are warranted.

At study endpoint, there was a significant reduction in the mean clinical score in treated versus untreated groups, and the lowest clinical score was noted in the 0.1% Fungisome-treated group in both models; however, the difference was not statistically significant.

In concordance with the clinical signs, the fungal corneal infiltration in both models also showed a reduction in treated versus untreated groups, although not statistically significant. Interestingly, a reduction in infiltration was noted earlier than improvement in the mean clinical score, indicating that monitoring the degree of infiltration is a better indicator of response to treatment. Mean size of corneal infiltration was lower in the 0.1% Fungisome- versus 0.1% AMB-treated groups in both models; however statistical significance was not seen. Thus, the findings could be attributed to chance. Evaluation in a larger number of rabbits could provide statistically significant evidence. A better clinical response with liposomal formulation has been reported by Habib et al.,^5^ who showed better efficacy of liposomal fluconazole than fluconazole against *C. albicans*-induced FK. A study evaluating the action of topically delivered 0.1% L-AMB in gel formula for treatment of fungal dermatophytic infections in humans also revealed better activity than conventional AMB.^24^

Our clinical findings were corroborated by microbiologic evidence as the total number of colonies recovered from infected (*A. flavus* and *C. albicans*) corneas was significantly lower in the 0.1% Fungisome-treated group compared to all other treated and untreated cases.

Assessment of toxicity revealed that conjunctival hyperemia in the lower fornix was higher in the AMB- compared to the 0.1% Fungisome-treated groups in both keratitis models. Although no statistical significance was achieved, this could indicate a higher toxicity of conventional versus liposomal preparation, which is expected as per the findings of previous studies.^10^,^28^ Although, it would have been better to use the uninfected contralateral eye to look for toxicity, unfortunately this was not done in the present trial. However, we assessed conjunctival congestion in the lower forniceal conjunctiva where we expect the drug to accumulate following topical administration. Additionally, assessment of circumcorneal congestion correlated with disease as per our observation while forniceal conjunctival congestion seemed to evolve independently. L-AMB has reduced ocular toxicity, as already shown for intravitreal injection in rabbits and rhesus monkeys.^29^ This is due to the localization of the drug inside the phospholipid bilayer limiting contact with epithelial cells, and the absence of deoxycholate. Although L-AMB is more expensive, has a shorter shelf life, and requires prior sonication, a combination of reduced toxicity and longer persistence in higher concentration at site of action would increase the therapeutic efficacy.

In conclusion, our study showed that topical application of Fungisome was more effective than no treatment. A trend of better and faster response with topical application of 0.1% Fungisome compared to 0.1% AMB was seen in this group of rabbits, but no statistical significance was achieved. Although limited by a small sample size and unclear generalizability to humans, this study suggested that Fungisome should be studied further as a potential antifungal agent for FK. To the best of our knowledge, this is the first in vivo study investigating the role of L-AMB compared to AMB in FK.
